# The impact of packed red blood cell transfusion on clinical outcomes in patients with septic shock treated with early goal directed therapy

**DOI:** 10.4103/0972-5229.76078

**Published:** 2010

**Authors:** Brian M. Fuller, Mithil Gajera, Christa Schorr, David Gerber, R. Phillip Dellinger, Joseph Parrillo, Sergio Zanotti

**Affiliations:** **From:** Department of Anesthesiology, Division of Critical Care Medicine, and the Division of Emergency Medicine, Washington University School of Medicine, St. Louis, MO, USA; 1 Department of Medicine, Divisions of Cardiovascular Disease and Critical Care Medicine, UMDNJ-Robert Wood Johnson Medical School at Camden, Cooper University Hospital, Camden, NJ, USA

**Keywords:** Early goal directed therapy, packed red blood cell, septic shock, severe sepsis, transfusion

## Abstract

**Background:**

The optimal hemoglobin level and transfusion threshold in patients with septic shock treated with an early, goal oriented approach to resuscitation remains unknown.

**Aims:**

To assess the impact of packed red blood cell (PRBC) transfusion on clinically relevant outcomes in patients with septic shock treated with early goal directed therapy (EGDT).

**Settings and Design:**

Retrospective cohort study of 93 patients who presented with septic shock, to a single center academic intensive care unit and received EGDT.

**Materials and Methods:**

Data were collected on patients identified via the Surviving Sepsis Campaign Chart Review database and linked to Project IMPACT database. The PRBC group and no PRBC group were compared by the Pearson chi-square and Fisher’s exact test to analyze statistical significance.

**Results:**

The PRBC group had a mortality of 41.2% vs. 33.9% in the no PRBC transfusion group (*P* = NS). The PRBC group also had more mechanical ventilation days (11.2 days vs. 5.0 days, (*P* ≤ 0.05), longer hospital length of stay (25.9 days vs. 12.5 days, (*P* ≤ 0.05), and longer intensive care unit length of stay (11.4 days vs. 3.8 days, (*P* ≤ 0.05).

**Conclusions:**

In this retrospective cohort study, transfusion of PRBCs was associated with worsened clinical outcomes in patients with septic shock treated with EGDT. Further studies are needed to determine the impact of transfusion of PRBC within the context of early resuscitation of patients with septic shock, as the beneficial effects gained by an early and goal oriented approach to resuscitation may be lost by the negative effects associated with PRBC transfusion.

## Introduction

The exact role of packed red blood cell (PRBC) transfusion in critically ill patients is largely unknown. Since the 1980s, PRBC transfusion has been scrutinized.[1] Mounting data now largely point to the deleterious effects of PRBC transfusion. It is difficult to determine which individual patient may benefit or be harmed from PRBC transfusion.

The average age of transfused PRBC in the United States is over 2 weeks.[2] Over this time frame, these stored cells undergo multiple changes that alter cellular morphology and physiology. A decrease in 2,3-diphosphoglycerate (2,3-DPG) occurs, increasing hemoglobin’s affinity for oxygen, which subsequently impairs offloading of oxygen. Morphologic changes include decreased PRBC size, increased friability, and decreased deformability, all of which result in altered rheology through the microcirculation. Negative clinical effects have also been associated with PRBC transfusion. These include infections, immunomodulation and immunosuppression, increased organ dysfunction and acute lung injury, as well as increased mortality.[3]

Early goal directed therapy (EGDT) for severe sepsis and septic shock is an algorithmic resuscitative approach aimed at reversal of sepsis induced tissue hypoperfusion.[4] The original study showed a significant improvement in mortality and these findings have been reproduced in multiple centers.[5] Despite the concerns with respect to altered cellular physiology, worsened clinical outcomes, and contrasting high level evidence,[6] EGDT recommends transfusion of PRBC to maintain a hematocrit of 30% or more in the face of perceived ongoing oxygen debt (mixed central venous oxygen saturation < 70%).

Significant amounts of data point to the harmful effects of PRBC transfusion, both in retrospective and observational studies, as well as large-scale clinical trials. Despite this, the optimal hemoglobin level and transfusion threshold in patients with septic shock treated with an early, goal oriented approach to resuscitation remains unknown. The aim of this study was to assess the impact of PRBC transfusion on clinically relevant outcomes in patients with septic shock treated with EGDT. Consistent with previous data, we found a deleterious impact of PRBC transfusion.

## Materials and Methods

**Figure 1:**
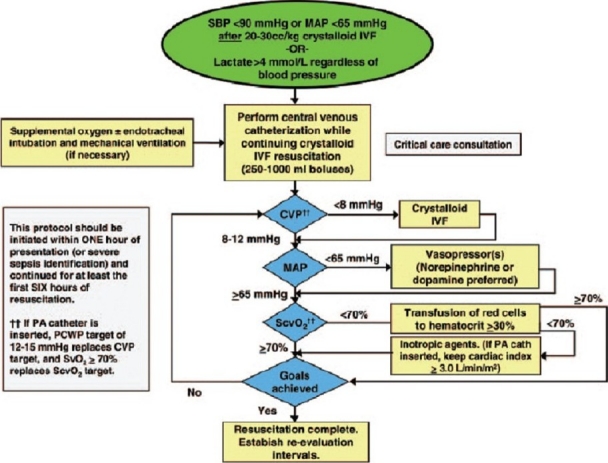
The early goal directed therapy protocol utilized at Cooper University Hospital

This single-center retrospective cohort study was conducted in a large, urban, academic teaching hospital, with an annual Emergency Department (ED) census of approximately 56,000 patients and a 30-bed medical–surgical intensive care unit (ICU). The study protocol was approved by the local institutional review board with waiver of informed consent. Data were collected on 93 consecutive patients who presented between February 2005 and May 2008 in septic shock and received EGDT. The trigger for EGDT at our institution is systolic blood pressure (BP) less than 90 mmHg or mean arterial pressure (MAP) less than 65 mmHg despite a crystalloid challenge of 20–30 ml/kg, or initial serum lactate concentration greater than 4 mmol/l [Figure 1]. For the purpose of this study, patients were divided into two groups: PRBC transfusion group and no PRBC transfusion group.

Data were collected on patients identified via the Surviving Sepsis Campaign Chart Review database and linked to Project IMPACT database. Primary data collection was done by two abstractors (MG and CS). CS has had extensive experience and training in database management and chart review. MG was trained in the data retrieval process prior to study initiation. Variables were defined prior to data extraction and placed in a standardized format during the data collection process. Regular meetings and monitoring of data collection were performed and the chart reviewers were blinded to study hypothesis. There was good inter-rater agreement between chart reviewers, though this was not statistically tested. The following data were collected with respect to PRBC transfusion group and no PRBC transfusion group: age, gender, race, Acute Physiology and Chronic Health Evaluation (APACHE II) score, total intravenous fluids (IVF) administered, first vasoactive medication given, initial lactate level, estimated time to central venous pressure goal (CVP 8ET), and achievement of central venous mixed oxygen saturation (ScvO_2_) >70%.

The primary outcome measure was hospitality mortality. Secondary outcomes included mechanical ventilation (MV) days, intensive care unit length of stay (ILOS), and hospital length of stay (HLOS). The PRBC group and no PRBC group were compared by the Pearson chi-square and Fisher’s exact test to analyze statistical significance. Statistical significance was defined as *P* ≤ 0.05.

## Results

A total of 93 patients were included in this study. 97% of patients in the PRBC group originated in the ED prior to ICU admission, compared with 94.9% of patients in the no PRBC group (*P* = NS). 34 patients received PRBC transfusion, with an average of 4.56 units per patient. There were no significant differences in baseline characteristics between the two groups [Table 1]. Average age was 63.5 years in the PRBC group and 59.3 in the no PRBC group (*P* = 0.199). There were no significant differences in gender or race distributions. Baseline APACHE II score was 21.1 in the PRBC group and 20.3 in the no PRBC group (*P* = 0.682). Initial lactate was 6.0 and 5.4, respectively (*P* = 0.463). With respect to resuscitation variables [Table 2], there was no significant difference in time to achievement of CVP of 8 (*P* = 0.135) or ScvO_2_ of 70% (*P* = 0.301). In the patients requiring vasoactive medications, norepinephrine was chosen as the first line vasoactive medication in 81.3% of patients in the PRBC group and in 83.8% of the no PRBC group (*P* = 0.770). Patients receiving PRBC transfusion did, however, receive more intravenous fluids over the first 6 hours (*P* ≤ 0.05) and first 72 hours (*P* ≤ 0.05). There was no difference in estimated time to antibiotics between the two groups: 165 minutes in the PRBC group and 147 minutes in the no PRBC group (*P* = NS).

**Table 1:** Baseline characteristics

Variable	PRBC (n = 34)	No PRBC (n = 59)	*P* value
Age (years)	63.5	59.3	0.199
Gender			
Male	22 (64.7)	33 (55.9)	0.512
Female	12 (35.3)	26 (44.1)	
Race			
Black	15 (44.1)	22 (37.3)	0.676
Hispanic	3 (8.8)	9 (15.3)	
White	16 (47.1)	27 (45.8)	
Other	0 (0)	1 (1.7)	
APACHE II	21.1	20.3	0.682
Lactate (mmol/l)	6.0	5.4	0.463

PBRC: Packed red blood cell, Values in paranthesis indicates percentage

**Table 2:** Resuscitation variables

Variable	PRBC (*n* = 34)	No PRBC (*n* = 59)	*P* value
Intravenous fluids (l)			
0–6 hours	5.7	3.9	<0.05
6–72 hours	17.6	13.0	<0.05
Total	23.3	16.9	<0.05
First vasoactive med.			
Norepinephrine	26 (81.3)	31 (83.8)	0.770
Dopamine	5 (15.6)	4 (10.8)	
Dobutamine	1 (3.1)	2 (5.4)	
CVP 8ET (minutes)	732.0	465.3	0.135
ScvO2			
70% after 24 hours	5 (15.6)	4 (7.1)	0.301
70% within 24 hours	23 (71.9)	37 (66.1)	
Not obtained	4 (12.5)	14 (25.0)	
Average PRBCs (units)	4.56	0	<0.05

PBRC: Packed red blood cell, Values in paranthesis indicates percentage

Patients receiving PRBC transfusion had a hospital mortality of 41.2% compared with a hospital mortality of 33.9% in the no PRBC group (*P* = NS) [Table 3].There was a significant difference between the PRBC group and no PRBC group with respect to MV days (11.2 vs. 5.0, (*P* ≤ 0.05), ILOS (11.4 days vs. 3.8 days, (*P* ≤ 0.05), and HLOS (25.9 days vs. 12.5 days, (*P* ≤ 0.05). Also, in the subgroup of patients transfused within the first 24 hours of their hospital stay (*n* = 14), there was a slight difference in hospital mortality (35.7%, *P* = NS) and greater MV days (7.2 days, *P* = NS), while there was a significant increase in ILOS (7.4 days, *P* < 0.05) and HLOS (21.1 days, (*P* ≤0.05).

**Table 3:** Clinical outcomes

Variable	PRBC (*n* = 34)	No PRBC (*n* = 59)	*P* value
MV	25 (73.5)	23 (39.0)	<0.05
Days	11.2	5.0	
ILOS (days)	11.4	3.8	<0.05
HLOS (days)	25.9	12.5	<0.05
Hospital mortality no.	14 (41.2)	20 (33.9)	0.510

PBRC: Packed red blood cell, Values in paranthesis indicates percentage

## Discussion

In this study, 93 patients with septic shock and receiving EGDT were studied. Patients receiving PRBC transfusion (*n* = 34) had worse clinical outcomes compared with those patients not receiving PRBC transfusion. These data are consistent with previous trials demonstrating the deleterious effects of PRBC transfusion.

Anemia in critical illness is common, and the majority of patients admitted to the ICU become anemic by day 3 of their ICU stay.[7] Compensatory mechanisms, such as increased cardiac output and increased oxygen extraction ratio, dictate that anemia is normally well tolerated in healthy individuals. Unfortunately, this is not the case in the critically ill and published data point to worse outcomes in these patients.[8]

With the recognition of the deleterious effects of anemia, the simple solution seems to be to correct this anemia with PRBC transfusion. Unfortunately, the majority of data states that this simple solution of fixing anemia with PRBC transfusion is likely to be harmful. Consistent data regarding PRBC transfusion point to worsened clinical outcomes, such as infection,[9] prolonged mechanical ventilation,[10] development of acute respiratory distress syndrome (ARDS)[11] and ventilator associated pneumonia,[12] as well as mortality.[13–15] While many of these studies are individually flawed based on their retrospective nature, the results taken as a whole are very consistent: the transfusion of PRBC is not a benign intervention.

Despite these concerns, EGDT calls for an increase in hematocrit to at least 30% in the setting of perceived tissue oxygen deficit.[4] This is based on the physiologic rationale of anemia in the setting of potential delivery dependant oxygen consumption.[5] Unfortunately, due to changes occurring at the cellular level during PRBC storage, there may be equally compelling physiologic rationale to not transfuse PRBC in the early stages of septic shock.[16]

The current study has several limitations. The small sample size makes drawing conclusions difficult. The retrospective design has inherent limitations. Although we capture a robust amount of data involving septic patients at our institution, we cannot exclude the possibility of unaccounted or missing data, which may cause undetected differences in baseline characteristics. For example, the PRBC group received higher volumes of intravenous fluids during their resuscitation. This could be reflective of a sicker baseline in these patients, though this seems somewhat less likely, given the similar APACHE scores, lactate levels, and catecholamine similarities. It is possible that the higher volume of fluids did affect MV days, favoring earlier extubation in the no PRBC group. Also, undetected treatment differences may have existed and these differences may have affected outcome.

A power analysis could not be conducted prior to the study, as the data used were what was available to the authors at the time. These facts, combined with the relatively small sample size in this trial, make drawing conclusions more difficult based on this trial alone. However, our data are consistent with large, prospective trials, as well as retrospective and observational studies in this arena.

Data concerning the negative effects of PRBC transfusion have been mounting over many years. Taken individually, many of the trials in this arena of critical care can be criticized for their limitations. Taken as a whole, the overwhelming body of evidence is consistent in raising concerns over the untoward effects of PRBC transfusion, and this has been corroborated by high-level, prospective data.[6]

In our study, the transfusion of PRBC was associated with deleterious effects on clinically relevant outcomes in septic shock patients. Enough concern exists that recommending a transfusion target in septic shock patients in the acute phase of resuscitation should be revisited. This concern exists not only to outcome, but also to the basic physiologic principles involving red cell changes and oxygen delivery and consumption as well. This trial should serve as hypothesis generating for future prospective trials in this arena, as it is indeed possible that the benefits of early resuscitation of septic shock patients may be lost by a liberal transfusion threshold.
